# Deep Learning-Based Recurrence Prediction in HER2-Low Breast Cancer: Comparison of MRI-Alone, Clinicopathologic-Alone, and Combined Models

**DOI:** 10.3390/diagnostics15151895

**Published:** 2025-07-29

**Authors:** Seoyun Choi, Youngmi Lee, Minwoo Lee, Jung Hee Byon, Eun Jung Choi

**Affiliations:** 1 Department of Radiology, Research Institute of Clinical Medicine of Jeonbuk National University-Biomedical Research Institute of Jeonbuk National University Hospital, Jeonbuk National University Medical School, Jeonju 54907, Republic of Korea; 2 Department of Statistics, Institute of Applied Statistics, Jeonbuk National University, Jeonju 54896, Republic of Korea; 3 Department of Statistics, Jeonbuk National University, Jeonju 54896, Republic of Korea; 4 Research Institute of Radiology, University of Ulsan College of Medicine, Ulsan University Hospital, Ulsan 44033, Republic of Korea

**Keywords:** breast neoplasm, human epidermal growth factor receptor 2, deep learning, recurrence, prediction algorithms

## Abstract

**Background/Objectives:** To develop a DL-based model predicting recurrence risk in HER2-low breast cancer patients and to compare performance of the MRI-alone, clinicopathologic-alone, and combined models. **Methods:** We analyzed 453 patients with HER2-low breast cancer who underwent surgery and preoperative breast MRI between May 2018 and April 2022. Patients were randomly assigned to either a training cohort (*n* = 331) or a test cohort (*n* = 122). Imaging features were extracted from DCE-MRI and ADC maps, with regions of interest manually annotated by radiologists. Clinicopathological features included tumor size, nodal status, histological grade, and hormone receptor status. Three DL prediction models were developed: a CNN-based MRI-alone model, a clinicopathologic-alone model based on a multi-layer perceptron (MLP) and a combined model integrating CNN-extracted MRI features with clinicopathological data via MLP. Model performance was evaluated using AUC, sensitivity, specificity, and F1-score. **Results:** The MRI-alone model achieved an AUC of 0.69 (95% CI, 0.68–0.69), with a sensitivity of 37.6% (95% CI, 35.7–39.4), specificity of 87.5% (95% CI, 86.9–88.2), and F1-score of 0.34 (95% CI, 0.33–0.35). The clinicopathologic-alone model yielded the highest AUC of 0.92 (95% CI, 0.92–0.92) and sensitivity of 93.6% (95% CI, 93.4–93.8), but showed the lowest specificity (72.3%, 95% CI, 71.8–72.8) and F1-score of 0.50 (95% CI, 0.49–0.50). The combined model demonstrated the most balanced performance, achieving an AUC of 0.90 (95% CI, 0.89–0.91), sensitivity of 80.0% (95% CI, 78.7–81.3), specificity of 83.2% (95% CI: 82.7–83.6), and the highest F1-score of 0.55 (95% CI, 0.54–0.57). **Conclusions:** The DL-based model combining MRI and clinicopathological features showed superior performance in predicting recurrence in HER2-low breast cancer. This multimodal approach offers a framework for individualized risk assessment and may aid in refining follow-up strategies.

## 1. Introduction

Understanding the biological and clinical significance of human epidermal growth factor receptor 2 (HER2) expression in breast cancer has profoundly influenced treatment [1]. HER2-positive breast cancers are especially aggressive and have a poor prognosis, leading to various treatment trials specifically targeting HER2-positive breast cancer [2]. Since the early 2000s, HER2 targeted therapy has significantly improved patient outcomes. However, several recent studies have identified limitations in the traditional binary classification of HER2 status as outlined in the 2022 guidelines of the American Society of Clinical Oncology/College of American Pathologists (ASCO/CAP). Particularly, antibody–drug conjugates, as a new therapy, have shown efficacy in patients with HER2-low (HER2 2+ without amplification and HER2 1+) tumors, previously classified as HER2-negative as demonstrated in the DENSITY-Breast04 trial [3,4]. Although HER2-low has recently gained clinical attention as a predictive biomarker for antibody–drug conjugate therapy, the diagnostic definition remains aligned with the 2018 ASCO/CAP guidelines, as no official update to HER2 testing methodology has been issued since [5].

The introduction of these new therapies has generated significant interest in HER2-low breast cancer, a distinct subtype with unique biological and clinical characteristics [6,7]. A recent study reported that patients with HER2-low breast cancer, like those with HER2-positive breast cancer, exhibited an increased risk of brain metastasis compared to HER2-zero breast cancer, emphasizing the need for vigilant surveillance [8]. Moreover, HER2-low status has been associated with longer disease-free survival (DFS) and lower rates of pathological complete response than HER2-zero status, irrespective of the hormone receptor status [9]. Given these distinct clinical characteristics and the growing importance of HER2-low breast cancer, further research is warranted. In particular, studies that incorporate clinicopathologic variables, image data, and advanced artificial intelligence techniques could help refine the new classification of HER2 status and identify predictors of poor prognosis in HER2-low breast cancer. Notably, previous study has only identified tumor stage, lymphovascular invasion, Ki-67 index, and estrogen receptor expression levels as prognostic factors for prediction recurrence in HER2-low breast cancer. These findings have been based solely on clinicopathologic analyses or gene expression assays with limited integration of imaging data [10].

In this context, artificial intelligence, particularly deep learning (DL), has emerged as a promising tool that utilizes clinicopathologic variables for recurrence prediction and risk stratification in overall breast cancer [11,12,13,14,15]. In a recent systematic review, Silveira et al. highlighted the consistent superiority of DL-based models that integrate multimodal inputs, such as clinical, imaging, and histopathologic data, over conventional machine learning approaches in breast cancer recurrence prediction [16]. Likewise, Yao et al. proposed a multimodal DL model (ICSDA) combining histopathologic slides, gene expression, and clinical variables, achieving an AUC of 0.75 in external validation [17]. These findings underscore the value of combining complementary data sources to enhance prediction performance. While histopathologic and gene expression data offer valuable prognostic information, they typically require invasive procedures such as surgery or biopsy and are only available postoperatively. In contrast, radiologic imaging provides noninvasive and preoperative information, yet studies leveraging such data, particularly MRI, in HER2-low breast cancer remain scarce. In particular, no DL-based models have been developed specifically for predicting recurrence in HER2-low breast cancer by integrating both clinicopathologic variables and MRI features despite its growing clinical relevance.

Therefore, our study aimed to develop a DL-based model to predict recurrence risk in patients with HER2-low breast cancer and to compare the performance of the MRI-alone prediction model, clinicopathologic-alone prediction model, with combined MRI features and clinicopathologic variables. This approach was intended to facilitate the creation of personalized treatment plans and follow-up protocols tailored to this unique subtype.

## 2. Materials and Methods

### 2.1. Study Population

**Figure 1:**
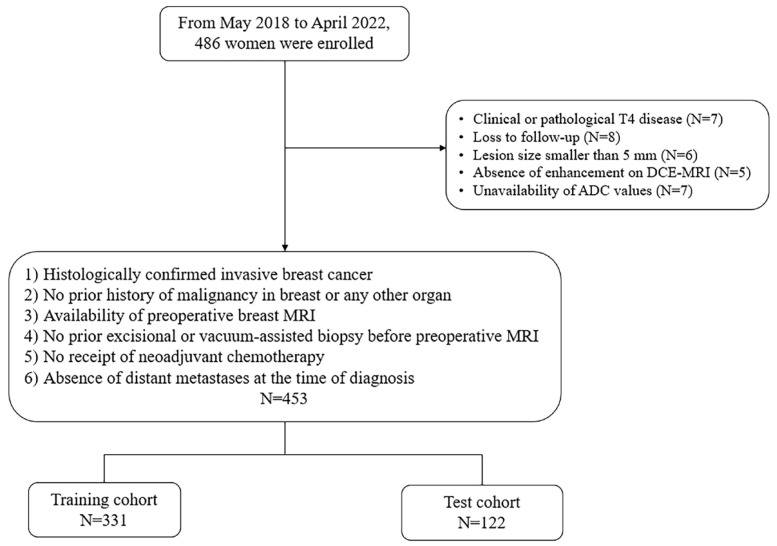
Flow chart of the inclusion and exclusion criteria of the study population.

This study was approved by our hospital’s institutional review board, which waived the requirement for informed consent. Between May 2018 and April 2022, consecutive female patients diagnosed with HER2-low breast cancer underwent curative surgery at our institution. Following surgery, adjuvant therapies—including radiation therapy, chemotherapy, and hormonal therapy—were administered based on individual patient conditions, as well as the clinical and molecular characteristics of the tumor. In total, 486 women were enrolled in this study (Figure 1). The inclusion criteria were as follows: (a) histologically confirmed invasive breast cancer, (b) no prior history of malignancy in the breast or any other organ, (c) availability of preoperative breast magnetic resonance (MR) imaging, (d) no prior excisional or vacuum-assisted biopsy before preoperative MR, (e) no history of neoadjuvant chemotherapy, and (f) absence of distant metastases at the time of diagnosis. A total of 33 patients were excluded for the following reasons: clinical or pathological T4 disease (*n* = 7), loss to follow-up (*n* = 8), lesion size < 5 mm (*n* = 6), absence of enhancement on dynamic contrast-enhanced MR imaging (*n* = 5), lack of apparent diffusion coefficient (ADC) values due to poor diffusion-weighted imaging (DWI) quality, or lack of diffusion restriction on the ADC map (*n* = 7). Ultimately, 453 lesions were included in this study. The patients were randomly allocated into two groups using statistical software: a training cohort (*n* = 331) and a test cohort (*n* = 122).

Breast cancer recurrence was classified as either locoregional (confined to the ipsilateral breast, chest wall, or regional lymph nodes, including the axillary, infraclavicular, or supraclavicular nodes [15]) or distant (metastases involving other anatomical sites). Follow-up assessments were conducted biannually during the first two years post-surgery and annually thereafter. Surveillance for locoregional recurrence included bilateral mammography, whole-breast ultrasonography, or breast MRI. To monitor distant metastases, imaging modalities, such as chest radiography, chest computed tomography (CT), bone scans, abdominal CT, and/or whole-body fluorine 18 fluorodeoxyglucose positron emission tomography, were used.

### 2.2. Clinicopathologic Features

Following a review of the patient medical records, data on cancer recurrence, patient age, tumor (T) stage, nodal (N) stage, multifocality, surgical margin status, presence of an extensive intraductal carcinoma (EIC) component, estrogen receptor (ER), progesterone receptor (PR), Ki-67, HER-2, histologic grade, and nuclear grade were collected. T and N stages were classified according to the American Joint Committee on Cancer (AJCC) staging system for breast cancer.

The presence of EIC was defined as intraductal carcinoma comprising > 25% of the total tumor volume in the final pathological assessment [18]. ER, PR, HER2, and Ki-67 expression levels were determined using immunohistochemistry (IHC). All HER-2 IHC scores were centrally evaluated by specialized breast cancer pathologists during core needle biopsy, following the 2018 American Society of Clinical Oncology (ASCO) and College of American Pathologists (CAP) guidelines for HER-2 testing [19]. Tumors were classified as ER- or PR-positive if at least 10% of the nuclei stained positively across 10 high-power fields. Ki-67 expression was categorized as either negative (<14%) or positive (≥14%) [20]. Breast cancer cases with an IHC score of 1+ or 2+ in combination with negative fluorescence in situ hybridization (FISH) results were classified as HER2-low [2]. Tumors were considered HER2-positive if IHC staining was 3+ or if the in situ hybridization (ISH) results were positive. Patients with HER2-positive tumors were excluded from the study.

### 2.3. Breast MRI Analysis

Two radiologists with 5 and 14 years of experience in interpreting breast MRIs independently reviewed the breast MRIs of the index lesions using the INFINITT PACS M6 version (INFINITT Healthcare Corp., Seoul, Korea) following a brief lecture and discussion on breast MRI interpretation to minimize interobserver variability. In cases of discrepancy between the two readers, a consensus was reached through joint review and discussion. No formal interobserver agreement statistics were calculated, but this consensus approach was used to ensure consistency in lesion interpretation and ROI annotation. They were blinded to all clinical information except for the presence of breast cancer. In cases of multifocal or multicentric disease, the largest focus or mass was selected for evaluation. Radiologists analyzed the MRI features of the index cancer according to the fifth edition of the Breast Imaging Reporting and Data System (BI-RADS) MRI lexicon [21]. The following characteristics were assessed: background parenchymal enhancement (BPE, minimal/mild or moderate/marked), shape (oval/round or irregular), margin (circumscribed, irregular, or spiculated), presence of combined non-mass enhancement (NME) adjacent to index breast cancer, and internal enhancement (homogeneous, heterogeneous, or rim). For kinetic analysis, time-intensity curves of the index cancers were retrospectively generated by each radiologist using dedicated DCE-MRI software (mean curve: Siemens Healthcare, Erlangen, Germany; syngo MR B17 version) or a computer-aided detection (CAD) system (CADstream, version 6.0, Confirma, Merge Healthcare Inc., Chicago, IL, USA). A region of interest (ROI) was placed over the area of the fastest enhancement or the most suspicious washout curve pattern of the lesion. The initial enhancement patterns were classified as slow, medium, or rapid, whereas the kinetic curve patterns were categorized as persistent, plateau, or washout [21]. To assess the DWI characteristics, radiologists placed ROIs on ADC maps, guided by the lesion extent, as observed on DCE-MRI. ADC values were computed on a pixel-by-pixel basis using the following equation: ADC = −ln [S_0_/S_1_]/b1, where S_0_ and S_1_ represent the signal intensities in the ROI obtained by two gradient factors, and b1 is 800 s/mm^2^. S_1_ corresponds to the signal intensity at the first contrast-enhanced point, and S_0_ refers to the unenhanced signal intensity in the ROI. To derive two representative ADC values, the radiologists first identified the largest cross-section of the tumor on the ADC map and placed an oval or round ROI within the lesion, ensuring that the ROI was as large as possible. Tumor ADC values were obtained by positioning the ROI over the most visually hypointense region of each tumor, carefully avoiding necrotic areas, cystic components, and imaging artifacts, with reference to both DCE-MRI and T2-weighted images. Additionally, three ROIs were placed on the adjacent breast parenchymal tissue where the ADC appeared to be the highest to measure the mean ADC values, and the highest value was designated as the maximal peritumoral ADC. The peritumoral-tumoral ADC ratio was calculated as the peritumoral maximal ADC divided by the tumoral ADC [22]. In cases of discordance between the radiologists’ assessments of the MRI features, a consensus was reached.

### 2.4. Breast MRI Examination Technique

All breast MRI examinations were performed using a 3.0-T system (Verio or Skyra; Siemens Healthcare, Erlangen, Germany) equipped with a dedicated breast coil. Dynamic contrast-enhanced MRI (DCE-MRI) was performed using a volumetric interpolated breath-hold examination (VIBE) sequence in conjunction with spectral adiabatic inversion recovery (SPAIR). The imaging parameters for DCE-MRI on the 3.0-T Skyra scanner included a repetition time (TR)/echo time (TE) of 4.5/1.6 milliseconds (ms), a matrix size of 448 × 381, a field of view (FOV) of 360 × 360 millimeters (mm), a flip angle of 10°, and a slice thickness of 1 mm without interslice gaps. For the 3.0-T Verio scanner, the parameters were TR/TE of 4.3/1.6 ms, matrix of 448 × 354, FOV of 340 × 340 mm, flip angle of 6°, and slice thickness of 1 mm without interslice gaps.

Each DCE-MRI examination consisted of one pre-contrast and five post-contrast dynamic series, acquired immediately following the intravenous administration of gadobutrol (Gadovist; Schering AG, Berlin, Germany) at a dose of 0.1 millimole (mmol) per kilogram (kg) body weight. The contrast agent was delivered as a bolus injection at a rate of 3 millimeters (mL) per second (s), followed by a 20-mL saline flush, using an automated injector. Standard subtraction images were generated by subtracting the pre-contrast images from the second dynamic (early peak) post-contrast images on a pixel-by-pixel basis. Additionally, maximum intensity projection (MIP) reconstruction was applied to the subtracted images.

Before contrast administration, DWI was performed using readout-segmented echo-planar imaging with the following parameters: TR/TE, 5200/53 ms; FOV, 340 × 205 mm; matrix size, 192 × 116; slice thickness, 4 mm; and total acquisition time, 2 min and 31 s, utilizing five readout segments. DWI acquisition incorporated *b*-values of 0 and 800 s/mm^2^.

### 2.5. Region of Interest Annotation and Preprocessing

To enhance the model’s predictive performance, we adopted a lesion-focused preprocessing strategy that emphasized diagnostically relevant regions rather than using full-size MR images. The MRI data, including five-phase DCE-MRI sequences and ADC maps of HER2-low breast cancer lesions, were initially obtained in DICOM format. The CNN model was trained using these cropped lesion patches, with one representative slice selected from each dynamic phase (five in total), forming a five-channel input per case. Each lesion was manually annotated by radiologists using bounding boxes to define ground truth regions, and cropped lesion patches were extracted accordingly. Due to compatibility limitations with the annotation tool (LabelMe (version 5.2.1)), which does not support DICOM files, the images were converted to high-quality, lossless TIF format prior to segmentation. Although this conversion may have led to minor loss of metadata, it was a practical requirement for annotation. As the model was based on pixel-level image data, the potential impact of metadata loss was not specifically assessed.

After extracting the lesion features, the training dataset exhibited an imbalance between the recurrence and non-recurrence groups (1:8.74). To address this, oversampling was applied to balance the dataset at a 1:1 ratio, by randomly resampling each class with replacement and shuffling the combined dataset.

A total of 331 train data and 122 test data samples were used. There were no missing values. Additionally, to ensure comparability across clinicopathological variables, as the scales of the variables differed and their distributions were not necessarily identical, the Min-Max scaling method was applied to standardize the data.

**Figure 2:**
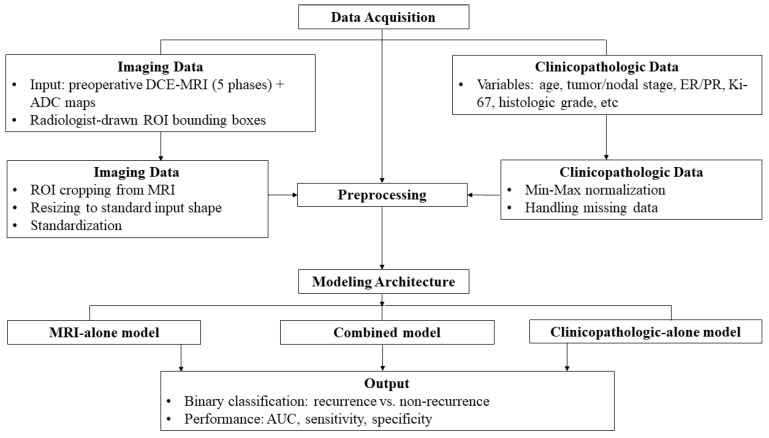
Overview of the proposed method for recurrence prediction in HER2-low breast cancer. The workflow comprises four major stages: (1) data acquisition, including preoperative MRI (DCE-MRI and DWI sequences) and clinicopathologic information such as tumor size, nodal status, histologic grade, and hormone receptor status; (2) feature extraction from MRI using convolutional neural networks (CNN) and from clinicopathologic data using a multilayer perceptron (MLP); (3) integration of image and non-image features into a combined representation; and (4) binary classification using a deep learning based model to predict recurrence risk. The final output is a recurrence vs. non-recurrence classification.

Figure 2 shows an overview flowchart of the proposed method, including image and clinicopathologic-MRI data acquisition, model architecture, and binary classification output.

### 2.6. Model Architecture and Feature Extraction

**Figure 3:**
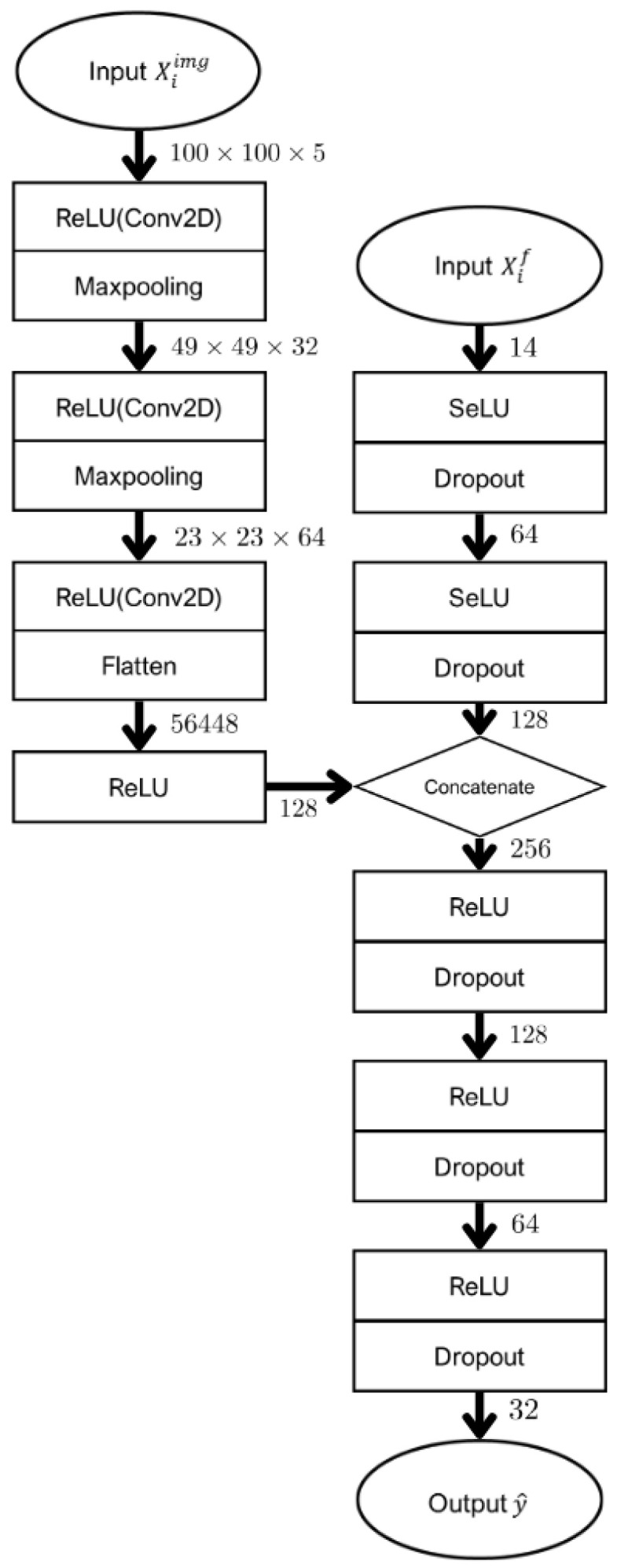
Proposed architectures. The model was designed to handle both image and non-image data. Non-image features were processed using a Multi-Layer Perceptron (MLP)-based architecture, while image features were extracted using Convolutional Neural Networks (CNNs).

The model was designed to handle both non-image and image data. Non-image features were processed using a multilayer perceptron (MLP)-based architecture, whereas image features were extracted using Convolutional Neural Networks (CNN) (Figure 3).

Let $X_{i}^{img}\in R^{100\times 100\times 5}$ denote the input MRI images and $X_{i}^{f}\in R^{14}\;$ represent the corresponding clinicopathologic features for the *i*-th patient, where $y_{i}\in \left\{0,1\right\}$ is the binary recurrence label. The model architecture comprises three main components: an image module, a feature module, and a combined feature module.

$$X_{i}^{img}\in R^{100\times 100\times 5},\;X_{i}^{f}\in R^{14},\;y_{i}\in \left\{0,1\right\},\;i=1,\ldots ,N$$

MRI images module:

The MRI image input passes through a series of convolutional and pooling operations as follows:

$$h_{1}^{img}=MaxPool2D\left(Relu\left(Conv2D\left(X_{i}^{img}\right)\right)\right)$$

$$h_{p}^{img}=MaxPool2D\left(Relu\left(Conv2D\left(h_{p-1}^{img}\right)\right)\right),\;p=2,3$$

$$h^{img}=Relu\left(flatten\left(h_{3}^{img}\right)\right)$$

Clinicopathologic features module:

Clinicopathological features were processed through fully connected layers with self-normalizing neural network (SELU) activation and dropout.

$$h_{1}^{f}=Dropout\left(Selu\left(X_{i}^{f}\right)\right)$$

$$h^{f}=Dropout\left(Selu\left(h_{1}^{f}\right)\right)$$

Combined features module:

The two latent representations are concatenated to form a combined representation:

$$h^{c}=\left[\begin{array}{c} h^{img}\\ h^{f} \end{array}\right]$$

This integrated vector is passed through a multi-layer perceptron:

$$h_{1}^{c}=Dropout\left(Relu\left(h^{c}\right)\right)$$

$$h_{q}^{c}=Dropout\left(Relu\left(h_{q-1}^{c}\right)\right),q=2,3$$

$${\hat{y}}_{i}=\sigma (h_{3}^{c})$$

Loss Function:

The model is optimized using binary cross-entropy loss:

$$L=-\frac{1}{N}\sum_{i=1}^{N}\left[y_{i}\ln\left(\hat{y_{i}}\right)+(1-y_{i})\ln\left(1-\hat{y_{i}}\right)\right]$$

The image data were processed through CNN layers, followed by max-pooling, after which they were flattened into a one-dimensional array and concatenated with the output from the non-image MLP layers. This ensured that both non-image and image features were fully represented within the model. The model that integrated both feature types was referred to as “Combined prediction model.” For comparison, a baseline model using only image features was developed, referred to as “MRI-alone prediction model.” Lastly, the model was implemented using Keras with TensorFlow backend (version 2.11.1).

### 2.7. Training and Evaluation

The prediction models were trained using binary cross-entropy as the loss function for the binary classification problem. The hyperparameters for epochs, patience, and batch size were set to 200, 10, and 16, respectively, for both prediction models. The validation data ratio within the training dataset was set to 0.3. The models were optimized using the Adam optimizer. The learning rates for the two prediction models were adjusted to ensure optimal fitting; the combined prediction model used a learning rate of 0.005, and the MRI-alone prediction model used a learning rate of 0.0005. The prediction models were independently trained 100 times to validate their generalizability, generating classification results under different random initializations and variations in the training data due to resampling during the oversampling process.

### 2.8. Statistical Analysis

The training and test cohorts were compared for both non-image data (clinicopathologic features) and image data (MRI features). For continuous variables, the Student’s *t*-test was used to assess the differences in means between groups, whereas for categorical variables, Pearson’s chi-squared test was used to evaluate the distribution. Additionally, a comparison between the recurrence and non-recurrence groups within the training cohort was performed using the same statistical tests to understand the characteristics of the dataset. Model performance was primarily evaluated using multiple metrics, including AUC, sensitivity, specificity, accuracy, and F1-score, to comprehensively assess the classification performance of the MRI-alone, clinicopathologic-alone, and combined models. To assess the reliability of the performance of the prediction model, the 95% confidence intervals (CIs) for these metrics were calculated using a t-distribution. All statistical analyses were conducted using Python (version 3.10.13), and the model design and fitting were implemented using the Keras package (version 3.0.0).

## 3. Results

Supplementary Materials present extended performance metrics and loss/accuracy curves supporting the main findings. 

### 3.1. General Characteristics of Patients

The histopathological diagnoses included 403 invasive ductal carcinomas, 29 invasive lobular carcinomas, 5 mixed ductal and lobular carcinomas, and 16 others, with no significant differences observed between the training and test cohorts. Among the clinicopathological variables, N stage and histological grade showed statistically significant differences between the training and test cohorts (Table 1).

Patient characteristics and MRI features based on recurrence status in the training cohort are presented in Table 2. Among the 331 eligible patients in the training cohort, 34 experienced recurrence, whereas 297 remained recurrence-free. Among the clinicopathological variables, patients who experienced recurrence were more likely to present with a higher T stage (26.2% vs. 50.0%, *p* = 0.001) and N stage (22.6% vs. 64.7%, *p* < 0.000) than those without recurrence. Additionally, multifocal tumors were significantly more frequent in the recurrence group (23.2% vs. 61.8%, *p* < 0.000) than in the non-recurrence group. Similarly, recurrence was more common among individuals with grade III histological features (33.7% vs. 70.6%, *p* < 0.000) and grade 3 nuclear features (18.5% vs. 50.0%, *p* < 0.000). PR negativity was also observed more frequently in patients with recurrence (41.2% vs. 17.8%, *p* = 0.003) compared to those without recurrence. In contrast, no statistically significant differences were identified in the ER status or Ki-67 index between the two groups.

In terms of MRI characteristics, DWI features, including ADC values, were not significantly different between the recurrence and non-recurrence groups. Similarly, BPE, mass shape, mass margin, non-mass enhancement (NME), internal enhancement, and kinetic curve types in the initial and late phases were not significantly different between the two groups.

### 3.2. Performance of the Model Predicting HER2-Low Breast Cancer Recurrence in the Training and Test Cohorts

In the training cohort, all three models—the MRI-alone, clinicopathologic-alone, and combined models—achieved excellent discrimination, each reaching an AUC of 0.99 (MRI-alone: 0.99, 95% CI: 0.99–1.00; clinicopathologic-alone and combined: 0.99, 95% CI: 0.99–0.99), as summarized in Table 3. However, performance differences became evident in the independent test cohort.

**Figure 4:**
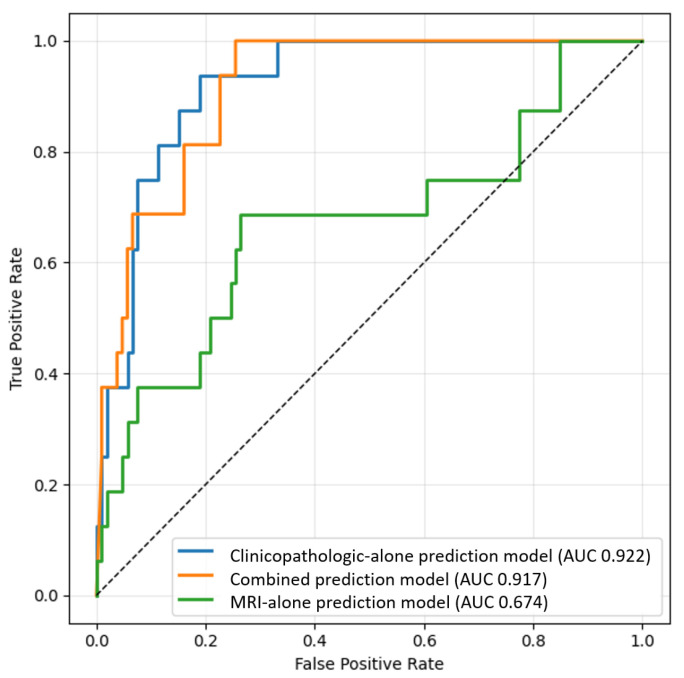
Comparison of prediction performance in test cohorts for the combined, MRI-alone, and clinicopathologic-alone prediction models. The median area under the curve (AUC) was 0.922 for the clinicopathologic-alone model, 0.91 for the combined model, and 0.68 for the MRI-alone model.

As illustrated in Figure 4 and detailed in Table 4, the clinicopathologic-alone model achieved the highest AUC of 0.92 (95% CI: 0.92–0.92), followed by the combined model at 0.90 (95% CI: 0.89–0.91), and the MRI-alone model at 0.69 (95% CI: 0.68–0.69). Despite the slightly higher AUC of the clinicopathologic-alone model, the combined model demonstrated superior overall balance across key performance metrics.

In the test cohort, the combined model achieved a sensitivity of 80.0% (95% CI: 78.7–81.3), specificity of 83.2% (95% CI: 82.7–83.6), F1-score of 0.55 (95% CI: 0.54–0.57), and the highest overall accuracy of 82.7% (95% CI: 82.4–83.1). The MRI-alone model showed the highest specificity (87.5%, 95% CI: 86.9–88.2), but had the lowest sensitivity (37.6%, 95% CI: 35.7–39.4) and F1-score (0.34, 95% CI: 0.33–0.35). Conversely, the clinicopathologic-alone model exhibited the highest sensitivity (93.6%, 95% CI: 93.4–93.8), and a moderate F1-score of 0.50 (95% CI: 0.49–0.50), but showed the lowest specificity (72.3%, 95% CI: 71.8–72.8) and accuracy (75.1%, 95% CI: 74.6–75.5) among the three models.

All performance improvements of the combined model over the MRI-alone and clinicopathologic-alone models were statistically significant (*p* < 0.001 for both comparisons; see Table 3 for training and Table 4 for test results).

**Figure 5:**
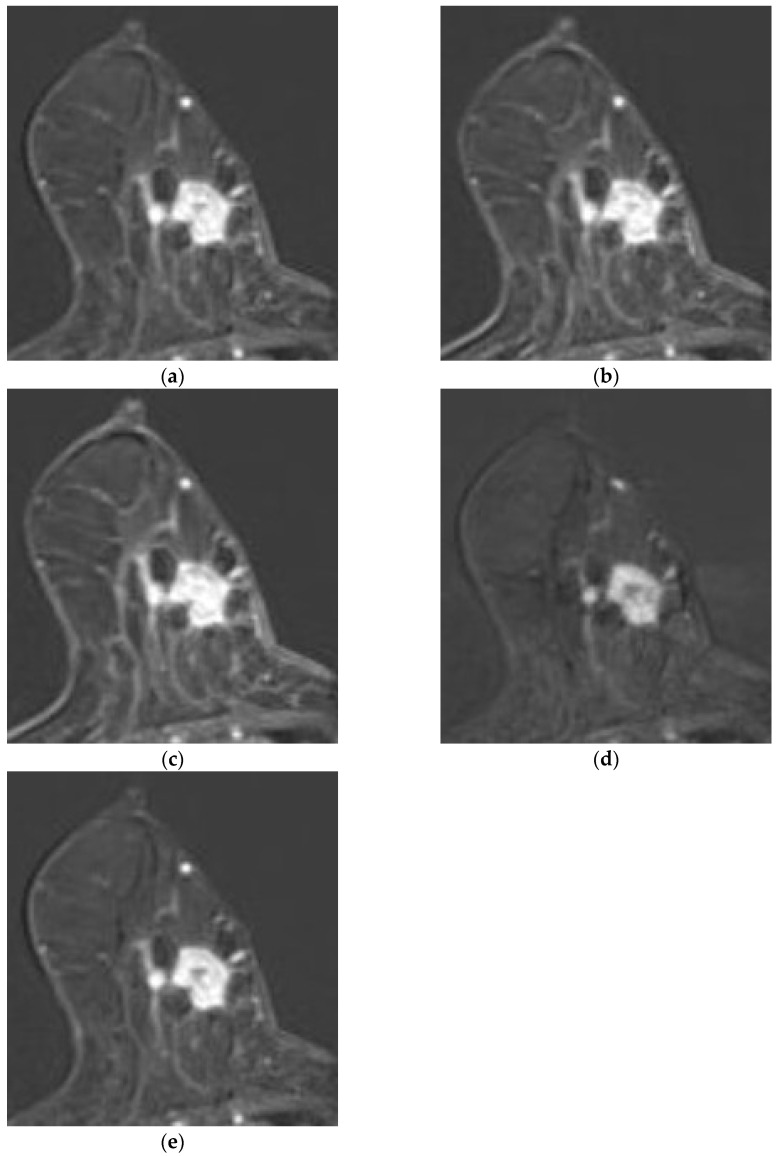
Representative case of HER2-low breast cancer with recurrence. The combined model correctly predicted recurrence, whereas the MRI-alone model did not. A 73-year-old woman with an almost entirely fatty breast and minimal background parenchymal enhancement (BPE). Axial contrast-enhanced T1-weighted MR images at (**a**) 1 min, (**b**) 2 min, (**c**) 3 min, (**d**) 4 min, and (**e**) 5 min post-contrast administration demonstrate a 1.4 × 1.7 cm irregular-shaped, irregular-margin mass with heterogeneous internal enhancement located in the right lower inner quadrant. The lesion exhibited rapid initial enhancement, followed by a plateau kinetic curve pattern. No axillary lymph node metastasis was identified. This recurrence case was not predicted by the MRI-alone deep learning model but was correctly predicted by the combined model integrating clinicopathological and MRI features.

**Figure 6:**
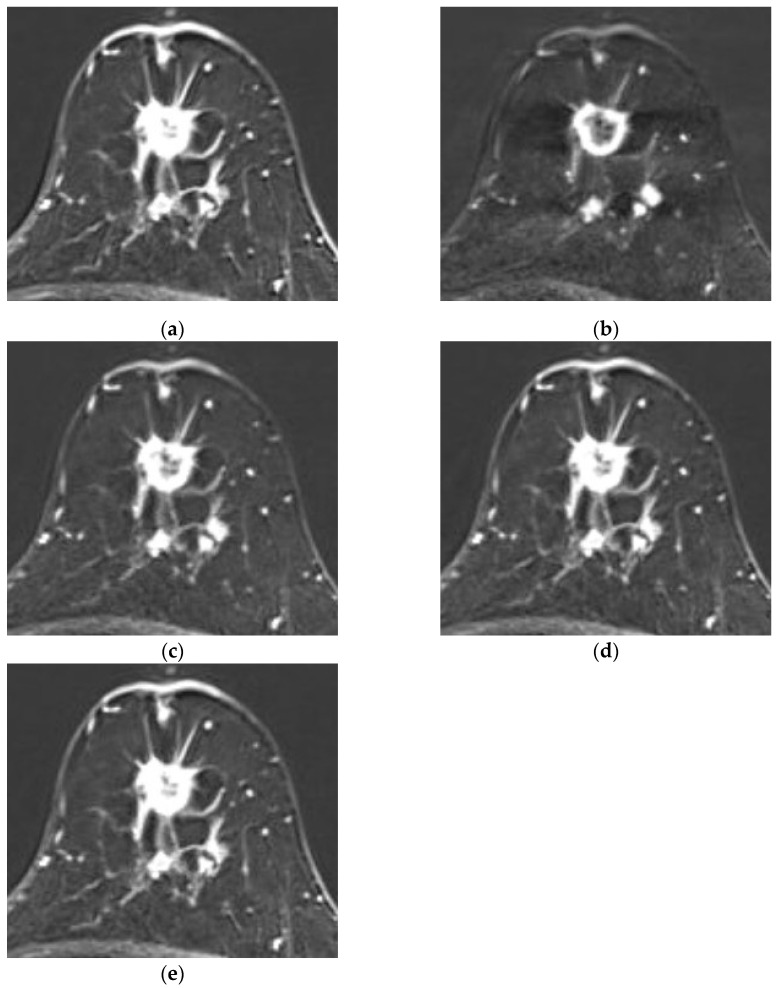
Representative case of HER2-low breast cancer with recurrence. Both the MRI-alone and combined models correctly predicted recurrence. A 50-year-old woman with heterogeneously dense fibroglandular tissue and moderate background parenchymal enhancement. Axial contrast-enhanced T1-weighted MR images at (**a**) 1 min, (**b**) 2 min, (**c**) 3 min, (**d**) 4 min, and (**e**) 5 min post-contrast administration show a 1.8 × 1.6 cm irregular-shaped, spiculated-margin, rim-enhancing mass located at the 12 o’clock position of the left breast. An additional 8 mm enhancing mass is seen posterior to the main lesion, with two other 8 mm enhancing masses laterally that appear interconnected. Although not shown in the images, another enhancing lesion was present in the upper inner quadrant (UIQ), confirming multicentric disease. Both models successfully predicted this recurrence case.

Representative recurrence cases are illustrated in Figure 5 and Figure 6. Figure 5 presents a case missed by the MRI-alone model but correctly predicted by both the combined and clinicopathologic-alone models, while Figure 6 depicts a multicentric recurrence case accurately identified by all three models.

## 4. Discussion

Recently, awareness of HER2-low breast cancer has surged significantly, following the DESTINY-Breast 04 study, which demonstrated the effectiveness of HER2-targeted therapy in patients with HER2-low metastatic breast cancer [23]. This growing recognition has led researchers to explore the biological distinctiveness of HER2-low breast cancer compared to HER2-zero and HER2-positive breast cancers [24,25]. Given these findings, the accurate recurrence prediction is important for optimizing follow-up protocols and treatment strategies for HER2-low breast cancer. To address this need, we developed an AI-model-based DL to predict recurrence, specifically for HER2-low breast cancer based on DL using clinicopathological variables and MRI features. Therefore, in this study, the AI model incorporating both combined clinicopathological variables and MRI features achieved a high AUC of 0.90 and demonstrated high sensitivity and specificity, outperforming the model using MRI-alone or clinicopathologic-alone for the prediction of recurrence of HER2-low breast cancer. These preliminary findings suggest that the AI model, using a combination of MRI features and clinicopathological variables, has the potential to predict recurrence in patients with HER2-low breast cancer.

Among the evaluated models, the MRI-alone prediction model demonstrated relatively lower performance (AUC of 0.69) in the test cohort, indicating moderate discriminatory ability and challenges in correctly identifying recurrent cases. A comparison with previous studies is necessary to better contextualize the performance of our model. Previous DL studies in broader or more aggressive breast cancer subtype have reported higher AUCs [18,26,27,28]. For instance, Yu et al. developed an MRI-based prediction model to predict prognosis in patients with triple-negative breast cancer (TNBC) prior to surgery, achieving an AUC of 0.88 in the validation cohort [12]. Similarly, Thawani et al. reported AUC values ranging from 0.7 to 0.81 for MRI-based recurrence prediction in patients treated with neoadjuvant chemotherapy [29]. A potential explanation for our model’s limited sensitivity may be related to class imbalance in the recurrence cases, accounting for approximately 13% of the total sample. This class imbalance may have hindered the model’s ability to effectively learn patterns that distinguish recurrence from non-recurrence, thereby contributing to a higher rate of false negatives. Additionally, MRI-based prediction models often rely heavily on imaging characteristics such as enhancement patterns, tumor morphology, and textural features. Although these features may correlate with tumor aggressiveness, they may not fully capture the complexity of recurrence risk factors, particularly in HER2-low breast cancer, where clinicopathological variables play a substantial role.

The clinicopathologic-alone model showed relatively high AUC values but demonstrated lower specificity and accuracy compared to the combined model. Clinically, a high AUC generally indicates robust overall discriminatory ability. Nonetheless, low specificity and accuracy imply a substantial risk of misclassification, potentially increasing false positive and false negative rate, unnecessary follow-up examinations, inefficient use of medical resources, reduced diagnostic reliability, and psychological stress for patients, as reported in previous studies [30,31]. In addition, the F1 score of the clinicopathologic-alone model was significantly lower than that of the combined model, indicating its limited ability to achieve a balanced trade-off between sensitivity and precision in recurrence prediction. Therefore, exclusive reliance on clinicopathologic variables could compromise clinical reliability and decision-making accuracy.

Accordingly, integrating radiologic and clinicopathologic data may improve predictive accuracy by enabling more comprehensive characterization of tumor heterogeneity in clinical practice. In this study, the combined model demonstrated balanced performance and achieved a high AUC of 0.90 in the test cohort, with favorable sensitivity (80.0%), specificity (83.2%), accuracy (82.7%), and F1 score (0.55), suggesting potential clinical utility. Notably, the combined prediction model did not exhibit signs of overfitting and maintained consistent performance in the test cohort, underscoring its robustness. These findings suggest that integrating both MRI features and clinicopathologic data enables more precise characterization of tumor biology and recurrence risk than using either modality alone. Consistent with our results, a previous study which focused on recurrence prediction in young women with breast cancer reported that the combined model using clinicopathologic variables and imaging features demonstrated high diagnostic performance [32]. Similarly, other previous studies reported high AUC using combined models with MRI features and clinicopathologic data for predicting pathologic complete response after neoadjuvant chemotherapy, highlighting the value of multimodal integration in personalized treatment planning [33,34]. Building on this evidence, our study is among the first to develop a recurrence prediction model tailored specifically for HER2-low breast cancer, with the combined model demonstrating balanced and high performance across multiple metrics. Developing a recurrence prediction model tailored to HER2-low breast cancer, a clinically distinct subtype, by combining imaging features and traditional prognostic markers represents a meaningful and novel contribution. The incorporation of MRI features into prediction models offers several practical advantages. As described in recent literature, predictive and prognostic breast MRI does not require additional patient procedures and utilizes routinely acquired preoperative imaging data, making it a cost-effective and noninvasive alternative to tissue-based biomarkers. This approach transforms breast MRI into a “one-stop shop” capable of providing both diagnostic and prognostic information. Furthermore, adding clinicopathological variables such as tumor size, nodal status, and histologic grade enhances the clinical applicability of the model [20]. Previous reviews of over 20 risk prediction models have shown that these traditional factors outperform genetic or biomolecular data alone in stratifying patients into meaningful risk categories [35].

This study had several limitations. First, this was a single-center retrospective analysis, which may limit the generalizability of the findings to a broader population. Second, although the dataset was divided into training and test cohorts, validation was performed using only internal data, serving as preliminary study. While the combined model achieved excellent performance in the internal test cohort, external validation is critical before the model can be considered for clinical implementation. Prior studies have emphasized that models validated only on internal datasets often overestimate generalizability and may fail to perform consistently across institutions with different imaging protocols and patient demographics [36,37]. To ensure robustness and clinical applicability, future studies should include external validation using large, multi-institutional datasets. Third, although all MR images were acquired from systems produced by the same vendor, they were obtained using two different MRI models. Subtle performance variations between the systems may have influenced the imaging quality and, consequently, model performance. Forth, the process of extracting imaging features relied on manual annotation of the ROI by radiologists, involving bounding-box delineation to localize tumor lesions. Although this step was carefully performed, the subjectivity inherent to manual segmentation may introduce variability, highlighting the need for the further development of automated or standardized lesion annotation techniques. Fifth, although oversampling was applied during training to address class imbalance (originally 1:8.74), this approach may have hindered the model’s ability to learn robust discriminative patterns and does not fully reflect the natural distribution of recurrent cases observed in clinical settings [38]. Finally, the combined prediction model has two notable limitations. The first limitation is that the five MRI slices were acquired sequentially across specific sequences and therefore possessed temporal characteristics. Because the image input is treated as a tensor of size 100 × 100 × 5, the CNN processes all five channels simultaneously and is unlikely to capture these sequence-level temporal dynamics. The second limitation relates to interpretability, which is a common challenge among many deep learning approaches. After the image and non-image features are concatenated, a fully connected layer produces the final representation, making it difficult to determine which specific features contribute the most to the classification outcome. To address this, methods such as Grad-CAM have been proposed to visualize pixel-level contributions in image inputs, and SHAP values have been used to quantify the importance of individual structured variables [39,40]. These post hoc interpretability tools may enhance transparency and clinical trustworthiness. Future research could integrate such techniques or develop tailored interpretability frameworks specifically for multimodal fusion models.

## 5. Conclusions

In conclusion, this study demonstrated that a DL-based prediction model integrating MRI features with clinicopathological variables can effectively predict recurrence in HER2-low breast cancer. We developed and compared three models, MRI-alone, clinicopathologic-alone, and combined models, and found that the combined prediction model consistently outperformed both single-source models. While the clinicopathologic-alone model achieved relatively high AUC, it showed lower specificity and accuracy, highlighting the additive prognostic value of MRI features. The combined model achieved performance comparable to or exceeding that of existing approaches. Although further prospective multicenter validation is required, these findings highlight the value of multimodal data integration in improving individualized risk assessment and clinical decision-making.

## Figures and Tables

**Table 1:** Patient characteristics and MRI findings.

Variables	Total (*n* = 453)	Training Cohort (*n* = 331)	Test Cohort (*n* = 122)	*p* Value
**Clinicopathologic**				
Age (years) ^†^	54.2 ± 11.5	53.7 ± 11.6	55.5 ± 11.1	0.158
Menopausal state				0.848
Pre	202 (44.6)	149 (45.0)	53 (43.4)	
Post	251 (55.4)	182 (55.0)	69 (56.6)	
Pathology result				0.863
IDC	403 (89)	296 (89.4)	107 (87.7)	
ILC	29 (6.4)	20 (6.0)	9 (7.4)	
Mixed	5 (1.1)	3 (0.9)	4 (3.3)	
Others	16 (3.5)	12 (3.6)		
T stage				0.098
1	325 (71.7)	245 (74.0)	80 (65.6)	
2–3	128 (28.3)	86 (26.0)	42 (34.4)	
N stage				0.013
0	322 (71.1)	242 (73.1)	80 (65.6)	
1–3	131 (28.9)	89 (26.9)	42 (35.4)	
Multifocality				0.059
Negative	318 (70.2)	242 (72.8)	77 (63.1)	
Positive	135 (29.8)	90 (27.2)	45 (36.9)	
Surgical margin				0.777
Negative	431 (95.1)	316 (95.5)	115 (94.3)	
Positive	22 (4.9)	15 (4.5)	7 (5.7)	
EIC				0.806
Negative	351 (77.5)	255 (77.0)	96 (78.7)	
Positive	102 (22.5)	76 (23.0)	26 (21.3)	
ER				0.579
Negative	62 (13.7)	43 (13.0)	19 (15.6)	
Positive	391 (86.3)	288 (87.0)	103 (84.4)	
PR				0.109
Negative	101 (22.3)	67 (20.2)	34 (27.9)	
Positive	352 (77.7)	264 (79.8)	88 (72.1)	
Ki67				1.000
Negative	329 (72.6)	247 (74.6)	82 (67.2)	
Positive	121 (26.7)	81 (24.5)	40 (32.8)	
Unknown	3 (0.7)	3 (0.9)	NA	
p53				0.917
Negative	81 (17.9)	61 (18.4)	20 (16.4)	
Positive	369 (81.5)	267 (80.6)	102 (83.6)	
Unknown	3 (0.7)	3 (0.7)		
Histologic grade				0.021
I/II	268 (59.2)	207 (62.5)	61 (50.0)	
III	185 (40.8)	124 (37.5)	61 (50.5)	
Nuclear grade				0.215
I/II	347 (76.6)	259 (78.2)	88 (82.1)	
III	106 (23.4)	72 (21.8)	34 (27.9)	
**MRI**				
BPE				1.000
Minimal/mild	239 (52.8)	175 (52.9)	64 (52.5)	
Moderate/marked	214 (47.2)	156 (47.1)	58 (47.5)	
Mass shape				0.110
Oval/round	74 (16.3)	48 (14.5)	26 (21.3)	
Irregular	379 (83.7)	283 (85.5)	96 (78.7)	
Mass margin				0.332
Circumscribed	63 (13,9)	42 (12.7)	21 (17.2)	
Irregular	305 (67.3)	229 (69.2)	76 (62.3)	
Spiculated	85 (18.8)	60 (18.1)	25 (20.5)	
Non-mass enhancement				0.516
Negative	368 (81.2)	266 (80.4)	102 (83.6)	
Positive	85 (18.8)	65 (19.6)	20 (16.4)	
Internal enhancement				0.172
Homogeneous	31 (6.8)	26 (7.9)	5 (4.1)	
Heterogeneous	341 (75.3)	242 (73.1)	99 (81.1)	
Rim	81 (17.9)	63 (19.0)	18 (14.8)	
Initial phase				0.697
Fast	407 (89.8)	299 (90.3)	108 (88.5)	
Medium/slow	46 (10.2)	32 (9.7)	14 (11.5)	
Late phase				0.893
Persistent/plateau	252 (55.6)	183 (55.3)	69 (56.6)	
Washout	201 (44.4)	148 (44.7)	53 (43.4)	
DWI				
Tumoral ADC ^†^ (10^−6^ mm^2^/s) ^†^	1035.8 (282.1)	1026.6 (284.3)	1057.8 (266.8)	0.072
Peritumoral maximal ADC ^†^ (10^−6^ mm^2^/s) ^†^	1599.0 (379.0)	1608.9 (387.5)	1572.2 (355.2)	0.475
Peritumoral-tumoral ADC ratio ^†^	1.6 (0.5)	1.6 (0.6)	1.5 (0.3)	0.357

Numbers in parenthesis are percentage. IDC = invasive ductal carcinoma, ILC = invasive lobular carcinoma, EIC = extensive intraductal carcinoma component, ER = estrogen receptor, PR = progesterone receptor, BPE = background parenchymal enhancement, DWI = diffusion-weighted imaging, ADC = apparent diffusion coefficient. ^†^ Data are mean (standard deviation).

**Table 2:** Patient characteristics and MRI findings in the training cohort according to recurrence status.

Variables	Non-recurrence (*n* = 297)	Recurrence (*n* = 34)	*p* Value
**Clinicopathologic**			
Age (years) ^†^	53.9 ± 11.3	51.8 ± 13.5	0.304
Menopausal state			0.884
Pre	131 (44.1)	18 (52.9)	
Post	166 (55.9)	16 (47.1)	
Pathology result			0.529
IDC	265 (89.2)	31 (91.2)	
ILC	17 (5.7)	3 (8.8)	
Mixed	3 (1)	0	
Others	12 (4)	0	
T stage			0.001
1	228 (73.8)	17 (50.0)	
2–3	69 (26.2)	17 (50.0)	
N stage			<0.000
0	230 (77.4)	12 (35.3)	
1–3	67 (22.6)	22 (64.7)	
Multifocality			<0.000
Negative	228 (76.8)	13 (38.2)	
Positive	69 (23.2)	21 (61.8)	
Surgical margin			<0.000
Negative	290 (97.6)	26 (76.5)	
Positive	7 (2.4)	8 (23.5)	
EIC			0.001
Negative	239 (80.5)	16 (47.1)	
Positive	58 (19.5)	18 (52.9)	
ER			0.097
Negative	35 (11.8)	8 (23.5)	
Positive	262(88.2)	26 (76.5)	
PR			0.003
Negative	53 (17.8)	14 (41.2)	
Positive	244(82.2)	20 (58.8)	
Ki67			0.069
Negative	235 (79.1)	12 (35.3)	
Positive	59 (19.9)	22 (64.7)	
Unknown	3 (0.0)	0	
p53			0.286
Negative	53 (17.8)	8 (23.5)	
Positive	241 (82.2)	26 (76.5)	
Unknown	3 (0.0)	0	
Histologic grade			<0.000
I/II	197 (66.3)	10 (29.4)	
III	100 (33.7)	24 (70.6)	
Nuclear grade			<0.000
I/II	242 (81.5)	17 (50.0)	
III	55 (18.5)	17 (50.0)	
**MRI**			
BPE			0.592
Minimal/mild	159 (53.5)	16 (47.1)	
Moderate/marked	138 (46.5)	18 (52.9)	
Mass shape			0.462
Oval/round	45 (15.2)	3 (8.8)	
Irregular	252 (84.8)	31 (91.2)	
Mass margin			0.432
Circumscribed	40 (13.5)	2 (5.9)	
Irregular	203 (68.4)	26 (76.5)	
Spiculated	54 (18.2)	6 (17.6)	
Non-mass enhancement			0.198
Negative	242 (81.5)	24 (70.6)	
Positive	55 (18.5)	10 (29.4)	
Internal enhancement			0.317
Homogeneous	25 (8.4)	1 (2.9)	
Heterogeneous	218 (73.4)	24 (70.6)	
Rim	54 (18.2)	9 (26.5)	
Initial phase			0.274
Fast	266 (89.6)	33 (97.1)	
Medium/slow	31 (10.4)	1 (2.9)	
Late phase			0.403
Persistent/plateau	167 (88.6)	16 (52.9)	
Washout	130 (11.4)	18 (52.9)	
DWI			
Tumoral ADC ^†^ (10^−6^ mm^2^/s) ^†^	987.1 (283.5)	981.9 (295.4)	0.470
Peritumoral maximal ADC ^†^ (10^−6^ mm^2^/s) ^†^	1607.7 (390.4)	1619.8 (366.5)	0.955
Peritumoral-tumoral ADC ratio ^†^	1.7 (0.6)	1.7 (0.5)	0.910

Numbers in parenthesis are percentage. IDC = invasive ductal carcinoma, ILC = invasive lobular carcinoma, EIC = extensive intraductal carcinoma component, ER = estrogen receptor, PR = progesterone receptor, BPE = background parenchymal enhancement, DWI = diffusion-weighted imaging, ADC = apparent diffusion coefficient. ^†^ Data are mean (standard deviation).

**Table 3:** Performance of the prediction models in the training cohort.

	MRI-alone Model (A)	Clinicopathologic-alone Model (B)	Combined Model (C)	*p* Value	
				A vs. C	B vs. C
**Sensitivity (%)**	99.9 (99.8, 100.0)	94.7 (94.0, 95.5)	99.2 (98.9, 99.4)	<0.001	<0.001
**Specificity (%)**	98.9 (98.7, 99.0)	82.9 (82.2, 83.5)	95.0 (94.3, 95.7)	<0.001	<0.001
**Accuracy (%)**	99.4 (99.3, 99.5)	88.8 (88.2, 89.4)	97.1 (96.7, 97.4)	<0.001	<0.001
**AUC**	0.99 (0.99, 1.00)	0.94 (0.94, 0.95)	0.99 (0.99, 0.99)	<0.001	<0.001
**F1-score**	0.96 (0.94, 0.97)	0.89 (0.89, 0.90)	0.82 (0.80, 0.84)	<0.001	<0.001

Note: Numbers in parentheses are the 95% confidence intervals. AUC = area under the receiver operating characteristic curve.

**Table 4:** Performance of the prediction models in the test cohort.

	MRI-alone Model (A)	Clinicopathologic-alone Model (B)	Combined Model (C)	*p* Value	
				A vs. C	B vs. C
**Sensitivity (%)**	37.6 (35.7, 39.4)	93.6 (93.4, 93.8)	80.0 (78.7, 81.3)	<0.001	<0.001
**Specificity (%)**	87.5 (86.9, 88.2)	72.3 (71.8, 72.8)	83.2 (82.7, 83.6)	<0.001	<0.001
**Accuracy (%)**	81.0 (80.5, 81.5)	75.1 (74.6, 75.5)	82.7 (82.4, 83.1)	<0.001	<0.001
**AUC**	0.69 (0.68, 0.69)	0.92 (0.92, 0.92)	0.90 (0.89, 0.91)	<0.001	<0.001
**F1-score**	0.34 (0.33, 0.35)	0.50 (0.49, 0.50)	0.55 (0.54, 0.57)	<0.001	<0.001

Note: Numbers in parentheses are the 95% confidence intervals. AUC = area under the receiver operating characteristic curve.

## Data Availability

The data presented in this study are not publicly available due to personal health information privacy policy and ethical restrictions, but de-identification data are available from the corresponding author on reasonable request.

## Supplementary Materials

The following supporting information can be downloaded at: https://www.mdpi.com/article/10.3390/diagnostics15151895/s1, Figure S1. Training and validation loss (top) and accuracy (bottom) curves for the combined model; Table S1. Performance of the prediction models using training and test cohorts with focal loss without oversampling; Table S2. Threshold-dependent performance of the combined model (focal loss, test set); Table S3. Threshold-dependent performance of the MRI-alone model (focal loss, test set); Table S4. Threshold-dependent performance of the clinicopathologic-alone model (focal loss, test set). References [41,42] are cited in the Supplementary Materials.

## Abbreviations

The following abbreviations are used in this manuscript:

HER2	Human epidermal growth factor receptor 2
DFS	Disease-free survival
DL	Deep learning
MIP	Maximum intensity projection
MR	Magnetic resonance
ADC	Apparent diffusion coefficient
DWI	Diffusion-weighted imaging
CT	Computed tomography
EIC	Extensive intraductal carcinoma
ER	Estrogen receptor
PR	Progesterone receptor
IHC	Immunohistochemistry
FISH	Fluorescence in situ hybridization
BI-RADS	Breast Imaging Reporting and Data System
BPE	Background parenchymal enhancement
NME	Non-mass enhancement
CAD	Computer-aided detection
ROI	Region of interest
MLP	Multilayer perceptron
CNN	Convolutional neural network
SELU	Self-normalizing neural network
CI	Confidence intervals
AUC	Area under the curve
TNBC	Triple-negative breast cancer
