# Supplementary material for: Deep Learning-Based Recurrence Prediction in HER2-Low Breast Cancer: Comparison of MRI-Alone, Clinicopathologic-Alone, and Combined Models
Source: Diagnostics (Basel). 2025 Jul 29;15(15):1895. doi: 10.3390/diagnostics15151895 (PMC12346550; doi:10.3390/diagnostics15151895)
Supplement: Supplementary file 1 [file diagnostics-15-01895-s001.zip › diagnostics-3713107-supplementary.pdf]

diagnostics

MDPI

Supplementary Materials

This appendix provides supplementary results to support and extend the findings presented in the main text. It includes the training and validation loss/accuracy curves of the combined model, as well as performance metrics from additional experiments using the focal loss function to address class imbalance.

Loss and Accuracy Curves of the Combined Model

To mitigate the risk of overfitting, early stopping (patience = 10) and dropout (rate = 0.4) were applied during model training. Figure S1 presents the training and validation loss and accuracy curves for the combined model. These curves indicate that the model achieved stable convergence without signs of excessive overfitting.

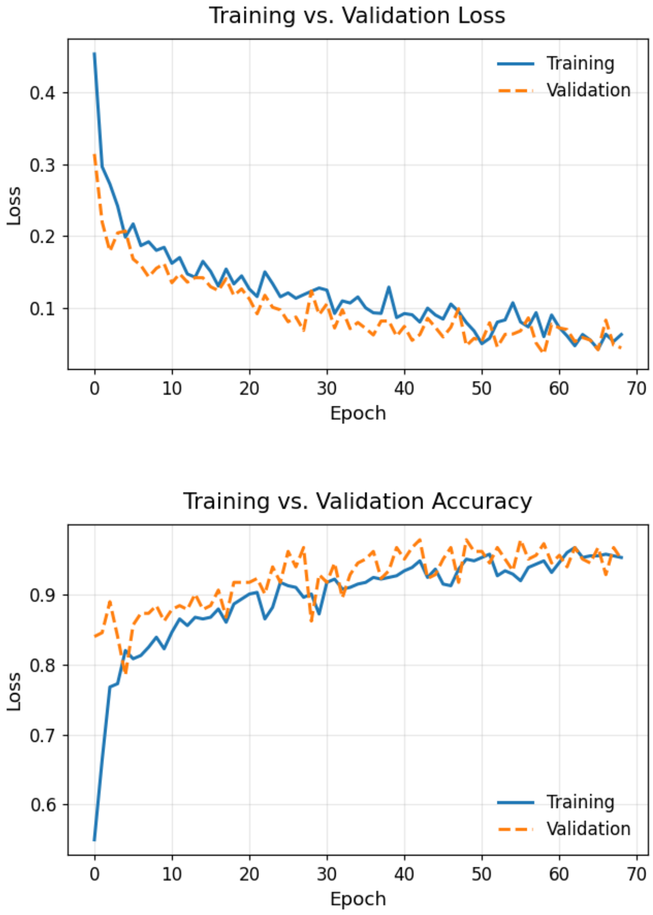

Figure S1. Training and validation loss (top) and accuracy (bottom) curves for the combined model.

Additional Results Using Focal Loss

To address the performance bias induced by the severe class imbalance in the training dataset, we conducted additional experiments using the focal loss function while preserving the original class distribution (i.e., without applying oversampling). Severe imbalance (e.g., 1:8.74 ratio of recurrence to non-recurrence) often leads conventional

 https://doi.org/10.3390/diagnostics15151895

loss functions such as binary cross-entropy to bias the model toward the majority class, resulting in poor sensitivity and under-detection of critical minority cases.

Focal loss is specifically designed to tackle this issue by down-weighting well-classified examples and focusing training on harder, misclassified cases. This dynamic weighting mechanism helps the model to learn more effectively from the underrepresented class without artificially augmenting the dataset, thereby reducing the risk of overfitting associated with oversampling strategies. This method has been shown to improve classification performance and calibration in numerous studies involving imbalanced medical data [41, 42].

Table S1 presents performance metrics—sensitivity, specificity, accuracy, and AUC—for the MRI-alone, clinicopathologic-alone and combined models under the focal loss setting. These results allow direct comparison with the main results trained with binary cross-entropy (Table 3 and 4).

Table S1. Performance of the prediction models using training and test cohorts with focal loss without oversampling.

	MRI-alone model		Clinicopathologic-alone model		Combined model	
	Training	Test	Training	Test	Training	Test
**Sensitivity**	100.0	17.0	89.0	95.2	99.9	54.5
**% (95%CI)**	(100.0, 100.0)	(14.8, 19.2)	(88.2, 89.7)	(94.7, 95.7)	(99.8, 100.0)	(51.8, 57.2)
**Specificity**	98.7	96.8	83.0	62.9	90.9	95.0
**% (95%CI)**	(98.5, 98.9)	(96.2, 97.3)	(82.6, 83.4)	(62.5, 63.3)	(89.6, 92.2)	(94.6, 95.4)
**Accuracy**	86.0	67.1	86.0	67.1	95.5	89.7
**% (95% CI)**	(85.5, 86.5)	(66.7, 67.5)	(85.5, 86.5)	(66.7, 67.5)	(94.8, 96.2)	(89.4, 90.0)
**AUC**	0.99	0.86	0.94	0.93	0.99	0.91
**(95% CI)**	(0.99, 0.99)	(0.86, 0.87)	(0.94, 0.94)	(0.92, 0.93)	(0.99, 0.99)	(0.91, 0.92)
**F1-score**	0.99	0.24	0.86	0.43	0.96	0.58
**(95% CI)**	(0.99, 0.99)	(0.21, 0.26)	(0.86, 0.87)	(0.43, 0.44)	(0.95, 0.96)	(0.56, 0.59)

Tables S2–S4 present threshold-dependent performance metrics for the combined, MRI-alone, and clinicopathologic-alone models respectively. For each threshold level, we report precision, F1-score, accuracy, and Brier score to show how performance varies across decision boundaries. These tables provide deeper insight into the models' classification behavior under varying operating points, focusing on metrics relevant to threshold selection.

Table S2. Threshold-dependent performance of the combined model (focal loss, test set).

Threshold	Precision (95% CI)	F1-score (95% CI)	Accuracy (95% CI)	Brier score (95% CI)
0.1	0.354 (0.337–0.371)	0.501 (0.489–0.513)	0.762 (0.748–0.776)	0.238 (0.224–0.252)
0.3	0.400 (0.383–0.418)	0.537 (0.526–0.548)	0.806 (0.795–0.817)	0.194 (0.183–0.205)

0.5	0.451 (0.429–0.473)	0.565 (0.554–0.576)	0.840 (0.832–0.848)	0.160 (0.152–0.168)
0.7	0.559 (0.538–0.579)	0.595 (0.580–0.610)	0.886 (0.881–0.890)	0.114 (0.110–0.119)
0.9	0.654 (0.619–0.688)	0.497 (0.466–0.529)	0.893 (0.889–0.898)	0.107 (0.102–0.111)

Table S3. Threshold-dependent performance of the MRI-alone model (focal loss, test set).

Threshold	Precision (95% CI)	F1-score (95% CI)	Accuracy (95% CI)	Brier score (95% CI)
0.1	0.296 (0.284–0.308)	0.357 (0.344–0.371)	0.782 (0.772–0.792)	0.218 (0.208–0.228)
0.3	0.353 (0.329–0.378)	0.301 (0.276–0.325)	0.840 (0.834–0.845)	0.160 (0.155–0.166)
0.5	0.483 (0.436–0.531)	0.257 (0.232–0.283)	0.865 (0.861–0.869)	0.135 (0.131–0.139)
0.7	0.516 (0.425–0.607)	0.173 (0.140–0.205)	0.872 (0.869–0.875)	0.128 (0.125–0.131)
0.9	0.315 (0.192–0.438)	0.060 (0.035–0.084)	0.872 (0.870–0.874)	0.128 (0.126–0.130)

Table S4. Threshold-dependent performance of the clinicopathologic-alone model (focal loss, test set).

Threshold	Precision (95% CI)	F1-score (95% CI)	Accuracy (95% CI)	Brier score (95% CI)
0.1	0.131 (0.131–0.132)	0.232 (0.232–0.233)	0.133 (0.132–0.135)	0.867 (0.865–0.868)
0.3	0.162 (0.160–0.165)	0.279 (0.275–0.283)	0.317 (0.304–0.331)	0.683 (0.669–0.696)
0.5	0.395 (0.392–0.399)	0.553 (0.455–0.438)	0.804 (0.801–0.807)	0.196 (0.193–0.199)
0.7	0.711 (0.694–0.728)	0.455 (0.438–0.472)	0.895 (0.893–0.897)	0.105 (0.103–0.107)
0.9	0.020 (0.000–0.048)	0.003 (0.000–0.008)	0.869 (0.869–0.869)	0.131 (0.131–0.131)
